# Comparison of percutaneous ablation and hepatectomy for liver metastasis: A single center retrospective study

**DOI:** 10.1002/cam4.6957

**Published:** 2024-02-01

**Authors:** Seiichi Tawara, Tetsuro Miyazaki, Ryosuke Kiyota, Yuki Maegawa, Takeshi Shimizu, Takuo Yamai, Shoichiro Kawai, Takuya Inoue, Hisateru Komatsu, Akira Tomokuni, Masaaki Motoori, Takayuki Yakushijin

**Affiliations:** ^1^ Department of Gastroenterology and Hepatology Osaka General Medical Center Osaka Japan; ^2^ Department of Gastrointestinal Surgery Osaka General Medical Center Osaka Japan

**Keywords:** colorectal, liver metastasis, liver metastasis ablation

## Abstract

**Aim:**

To investigate the current treatment for liver metastasis and clarify the indications for percutaneous thermal ablation for liver metastasis.

**Methods:**

Ninety‐two patients were enrolled and retrospectively analyzed. The patients underwent hepatectomy and/or percutaneous thermal ablation for liver metastases between January 2012 and December 2018. Twenty‐six patients who underwent ablation treatment and seven patients who underwent both ablation and hepatectomy were included in the ablation treatment group (group A). We compared these patients with 59 patients who underwent hepatectomy only (group H). Subgroup analyses were performed between ablation (group AC) for colorectal liver metastasis and hepatectomy (group HC) for colorectal liver metastasis in 17 and 53 patients, respectively.

**Results:**

The percentage of liver metastases other than colorectal cancer in group A was higher than that in the group H. Maximum tumor size in group A was significantly smaller than that in group H. Similarly, the patients in group AC were significantly older and demonstrated higher total bilirubin, lower serum albumin, and lower platelet counts than those in group HC. Overall survival was poorer in the AC group than that in the HC group. However, no differences were observed at metastasis ≤2 cm in size.

**Conclusions:**

Percutaneous thermal ablation was performed for many cancer types than hepatectomy. It is performed in elderly patients. We suggested that ablation for colorectal liver metastasis sized ≤2 cm is a suitable indication.

## 1 INTRODUCTION

Percutaneous thermal ablation for malignant tumors using radiofrequency or microwaves was developed in the late 20th century. Percutaneous radiofrequency ablation (RFA) has been used clinically since 1999 in Japan. Radiofrequency ablation for hepatocellular carcinoma was approved by the health insurance in 2004. It has become widely used in Japan as a general treatment strategy.^1^ Technologies supporting ablation, such as artificial pleural and ascitic fluid methods, contrast‐enhanced ultrasonography, and real‐time fusion imaging of ultrasonography with computed tomography (CT) or magnetic resonance imaging (MRI), have improved. Consequently, ablation is technically possible for most small hepatocellular carcinomas.^2^

However, the ablation of liver metastases is uncommon. It is well known that surgical treatment for colorectal cancer liver metastasis (CRLM) contributes to the patient's prognosis.^3^ A previous study has indicated that ablative therapy was inferior to resection in treating CRLM of >3 cm.^4^ Retrospective reports indicate that ablation for CRLM is inferior to resection.^5^ A randomized controlled trial comparing surgery and thermal ablation for CRLM is currently ongoing in Europe.^6^ However, the results have not yet been reported, and long‐term prognosis remains unclear. Therefore, ablation tends to be the second option for cases in which surgery cannot be performed. Because liver metastases in many types of cancer represent systemic metastases, local intervention for liver metastases tends to be avoided. Ablation of liver metastases other than colorectal cancer is not considered useful.

Surgery is an invasive procedure. Ablation does not require general anesthesia and has a lower incidence of complications than liver resection; therefore, it can be performed in patients with high surgical risk. There is no consistent opinion on the criteria for ablation adaptation, including the type of primary lesion, number and size of liver lesions, age, comorbidities, etc. This study aimed to investigate the treatment for liver metastasis at our institution and clarify the indications for percutaneous thermal ablation for liver metastasis.

## 2 PATIENTS AND METHODS

### 2.1 Patients

Ninety‐two patients with liver metastases were enrolled. The patients underwent consecutive hepatectomy and/or percutaneous thermal ablation for liver metastasis between January 2012 and December 2018 at the Osaka General Medical Center. In some patients who underwent ablation without liver tumor biopsy, liver metastasis was diagnosed using CT, MRI, PET, and tumor markers. These patients had no malignancies other than a treated primary cancer.

**FIGURE 1:**
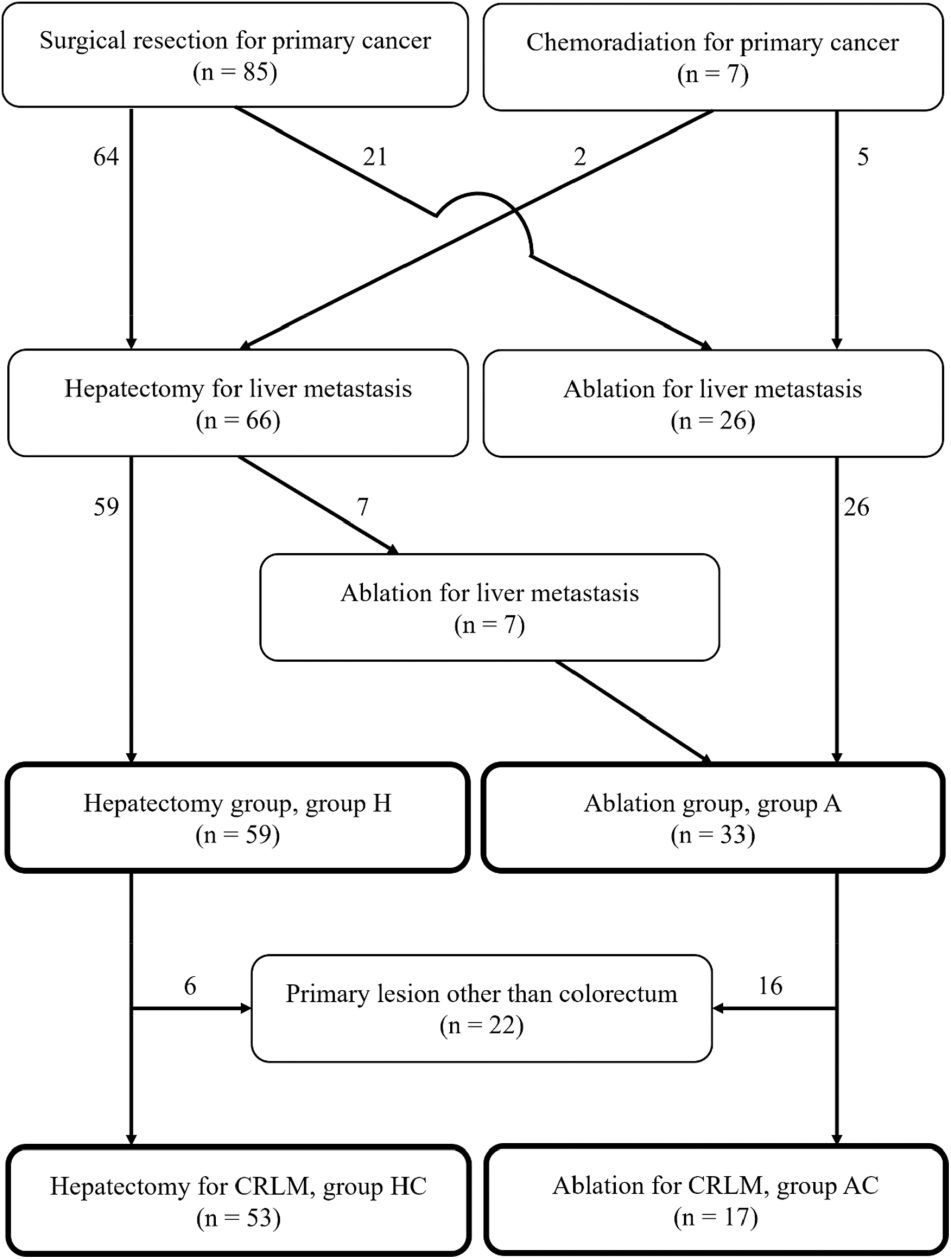
Flow chart of patient grouping in this study. This figure roughly illustrates the grouping of interventions. Some patients underwent several hepatectomy for multiple recurrences of liver metastases at different times. Similarly, some patients received multiple ablation treatments.

Eighty‐five patients underwent surgical resection and seven received chemoradiation therapy for their primary cancer as an initial treatment. During follow‐up, 66 patients underwent hepatectomy and 26 patients underwent ablation for liver metastases. Seven patients who underwent hepatectomy also underwent ablation for subsequent relapses. Twenty‐six patients who underwent ablation and seven who underwent both ablation and hepatectomy were included in the ablation group (group A). Fifty‐nine patients who underwent hepatectomy alone were included in the hepatectomy group (group H). Four patients who underwent several hepatectomies had multiple recurrences of liver metastases. Fourteen patients underwent multiple ablation procedures. Moreover, two subgroups were established from these groups by excluding cancers other than those of the colorectum: ablation (group AC) and hepatectomy (group HC) for colorectal liver metastasis (CRLM). Figure 1 illustrates the patient flow and grouping.

### 2.2 Indication and procedure of ablation and follow‐up examination

Percutaneous thermal ablation was adapted for patients with liver metastasis satisfying the following criteria: (i) ineligible for surgical resection or patient refusal for surgery; (ii) no vascular invasion; (iii) no other disease that determines the patient's prognosis; and (iv) the Child–Pugh scoring system is class A or B. Exclusion criteria were as follows: (i) tumor not visualized by ultrasonography and not accessible percutaneously; (ii) total bilirubin level ≥3.0 mg/dL; (iii) platelet count <5 × 10^4^/μL; (iv) prothrombin activity <50%; (v) refractory ascites; (vi) enterobiliary reflux; and (vii) adhesion between the tumor and gastrointestinal tract. In cases satisfying these conditions, ablation was performed in patients who were likely to have a possible cure or prolongation of life. Informed consent for ablation was obtained from all the patients.

Ablation procedures were performed percutaneously under ultrasound guidance (Aplio 500; Toshiba Medical Systems, Otawara, Tochigi, Japan; LOGIQ E9; General Electric Company, Boston, MA, USA). Artificial pleural effusion and ascites are used when required for tumor localization.^7^, ^8^ We administered local anesthesia to the skin and liver surface, and sufficient intravenous sedation. After adequate anesthesia, a 17‐gauge thermal ablation electrode (Cool‐Tip RF Ablation System; Covidien, Dublin, Ireland and VIVA RF system; STARmed Company, Goyang, South Korea) was inserted. The time required to energize each application ranged between 6 and 12 min. For large tumors, the electrode was inserted several times at different sites so that the entire tumor was necrotic. After the procedure, the patient remained in bed for 3 h.

A CT was performed 1–3 days after the ablation to evaluate the necrotic range. Complete ablation was defined as a low‐density area covering approximately 1 cm of the target tumor. For cases outside of this definition, the same procedure was repeated the following week. These cases were counted as single treatments, including additional ablation. Patients without operative complications were discharged a few days after the ablation. One month later, CT or MRI was performed again to evaluate the local viable lesions. Subsequently, imaging was performed every 3–6 months.

### 2.3 Statistical analysis

Statistical analysis was conducted using JMP Pro software (version 13.0; SAS Institute Inc., Cary, NC, USA). The Kruskal–Wallis and Wilcoxon tests were used to assess whether there were any significant differences between the groups. The Kaplan–Meier method was used to assess survival, and the groups were compared using the log‐rank test. Differences were considered statistically significant at *p* < 0.05. Data are reported as medians plus the entire range. The number of interventions and liver lesions per intervention are shown as averages because there were many one‐time interventions and single tumors.

## 3 RESULTS

### 3.1 Characteristics of each group in all cancers

Primary cancers in the enrolled patients in order of frequency were colorectal, gastric, esophageal, pancreatic, pharyngeal, lung, renal, breast, uterine, and renal cancers. The primary cancers in Group A were colorectal in 17 cases, gastric in seven cases, esophageal in three cases, and the pancreatic, pharyngeal, lung, renal, breast, and uterine in one case each. The primary cancers in Group H were colorectal in 53 cases; gastric in three cases; and the pancreatic, pharyngeal, and lung in one case each. Treatment for liver metastases other than colorectal cancer was 48% (16/33 cases) in group A, which was more frequent than 10% (6/59 cases) in group H (*p* < 0.0001) (Table 1). The median age of group A was 72 years, which was significantly higher than 67 years in group H (*p* < 0.05). There were no differences in sex or frequency of chemotherapy. Preoperative blood tests revealed significantly lower serum albumin levels (*p* < 0.0001) and platelet counts (*p* < 0.01) in group A.

**TABLE 1:** Demographic, clinical, and serological characteristics of each group in all cancers.

Factor	Group A	Group H	*p* Value
Age, year	72 [31–89]	67 [32–86]	<0.05
Gender, man/woman	21/12	36/23	0.80
Primary lesion, colorectum/others	17/16	53/6	<0.0001
Chemotherapy, yes/no	28/5	52/7	0.75
Maximum liver tumor diameter, mm	17 [8–40]	30 [10–160]	<0.0001
Number of liver lesions per intervention*	2.0 [1–6]	2.0 [1–12]	0.44
Number of liver interventions*	1.8 [1–5]	1.1 [1–3]	<0.0001
Total bilirubin, mg/dL	0.8 [0.2–3.3]	0.7 [0.3–1.1]	0.23
Serum albumin, g/dL	3.9 [3.0–4.7]	4.2 [2.7–4.8]	<0.0001
Prothrombin time, %	96.4 [70.9–114.4]	98.7 [75.7–125.0]	0.15
Platelet count, ×10^4^/μL	18.0 [9.0–36.8]	21.5 [13.6–46.0]	<0.01

* Items marked with asterisks are shown as averages. Otherwise, they are presented as numbers or medians. [−], numbers in brackets represent the entire range.

The median maximum tumor diameter was 17 mm (range, 8–40 mm) in group A and 30 mm (range, 10–160 mm) in group H. This was significantly higher in group H (*p* < 0.0001). No differences were observed in the number of liver metastases between the treatment groups. The average number of treatments was 1.8 in group A, which was more frequent than 1.1 in group H (*p* < 0.0001).

### 3.2 Characteristics of each group in CRLM

We analyzed the subgroups that included only CRLMs to clarify the effectiveness of ablation. In this analysis, excluding primary cancers other than colorectal cancer, 17 and 53 patients were assigned to the AC and HC groups, respectively (Figure 1, Table 2). The median age of the patients in group AC was 72, which was significantly 4 years older than that in group HC (*p* < 0.05). There were no differences in sex or frequency of chemotherapy. The median maximum tumor diameter was 16 mm (range, 8–31 mm) in the AC group and 30 mm (range, 10–160 mm) in the HC group. This was significantly higher in the group HC (*p* < 0.001). No significant differences were observed in the number of CRLM per treatment group. The average number of treatments was 1.9 (1–4 times) in the group AC, which was more frequent than 1.1 times (1–3 times) in the group HC (*p* < 0.0001). Patients in the group AC had significantly higher total bilirubin levels (*p* < 0.05), lower serum albumin levels (*p* < 0.05), and lower platelet counts (*p* < 0.01) than those in the group HC. Carcinoembryonic antigen levels were significantly higher in the group HC (*p* < 0.05).

**TABLE 2:** Demographic, clinical, and serological characteristics of each group in colorectal liver metastasis.

Factor	Group AC	Group HC	*p* Value
Age, year	72 [51–89]	68 [46–86]	<0.05
Gender, man/woman	10/7	32/21	0.91
Chemotherapy, yes/no	15/2	46/7	0.88
Maximum liver tumor diameter, mm	16 [8–31]	30 [10–160]	<0.001
Number of liver lesions per intervention*	2.4 [1–6]	2.1 [1–12]	0.20
Number of liver interventions*	1.9 [1–4]	1.1 [1–3]	<0.0001
Total bilirubin, mg/dL	0.8 [0.3–3.3]	0.7 [0.3–1.1]	<0.05
Serum albumin, g/dL	3.9 [3.4–4.6]	4.2 [2.7–4.8]	<0.05
Prothrombin time, %	96.5 [85.2–114.4]	101.0 [75.7–125.0]	0.58
Platelet count, ×10^4^/μL	16.6 [9.0–28.1]	22.3 [13.6–46.0]	<0.01
Carcinoembryonic antigen, ng/mL	3.8 [1.4–35.2]	7.5 [0.7–12221.2]	<0.05
Carbohydrate antigen 19–9, U/mL	8 [2–3613]	12 [2–27,771]	0.19
UICC stage at primary colorectal resection, I/II/IIIA/IIIB/IVA	2/1/4/1/9	4/8/8/4/29	0.79
T factor, 1/2/3/4a/4b	2/1/13/1/0	0/5/41/5/2	0.12
N factor, 0/1/2	5/10/2	22/23/8	0.54
M factor, 0/1a	8/9	24/29	0.90
Observation period after primary colorectal resection, day	1691 [792–3037]	2031 [280–4361]	0.12
Period from primary colorectal resection to liver intervention, day**	442 [66–1126]	264 [0–2289]	<0.05
Observation period after liver intervention, day	1003 [376–2971]	1813 [168–3489]	0.10

* Items marked with asterisks are shown as averages. Otherwise, they are presented as numbers or medians. [−], numbers in brackets represent the entire range.

** The period in group AC with hepatectomy is that time until the first ablation.

The median observation period after primary colorectal surgery was 2031 days in the HC group and 1691 days in the AC group. The period from primary colorectal resection to liver intervention was 264 and 442 days in the HC and AC groups, respectively. This duration was significantly longer in the AC group (*p* < 0.05). There were seven cases of simultaneous surgical resections of the liver and colorectum. In these cases, the period from primary colorectal resection to liver intervention was determined to be zero days. There were 15 cases in which planned chemotherapy for the reduction of CRLM and hepatectomy at the planned time were performed after primary colorectal resection in the HC group. However, ablation was performed for liver metastases that appeared during follow‐up. No scheduled ablation was performed before the primary surgery.

### 3.3 Overall survival of each group in CRLM

**FIGURE 2:**
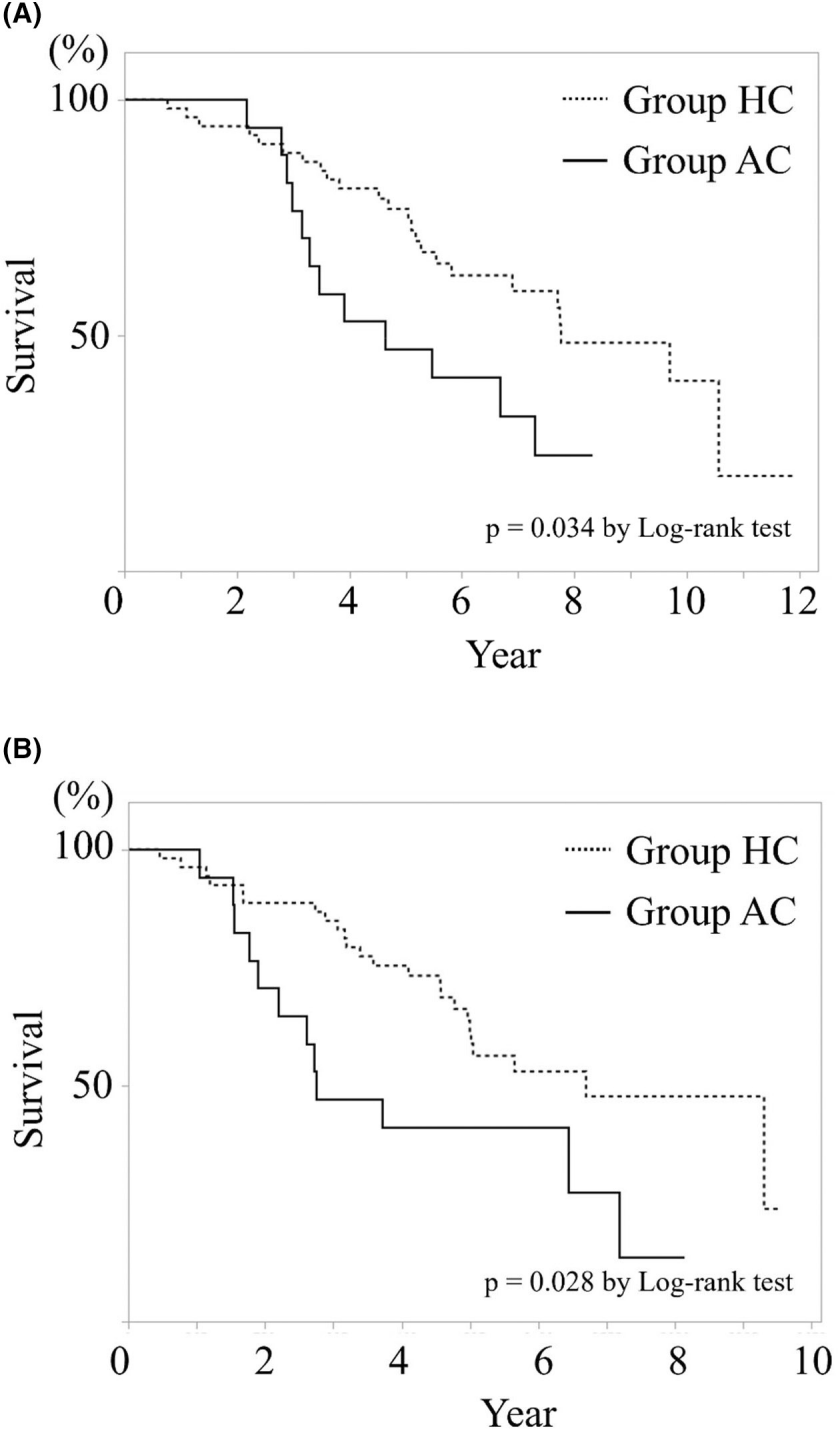
Overall survival after primary colorectal resection (A) and after liver intervention (B). Overall survival in the group HC both after primary colorectal resection and after liver metastasis treatment was significantly longer than those in the group AC.

The overall survival after primary colorectal resection in group HC was significantly longer than that in group AC (*p* = 0.034) (Figure 2A). The overall survival after hepatectomy or ablation in group HC was significantly longer than that in group AC (*p* = 0.028) (Figure 2B).

**FIGURE 3:**
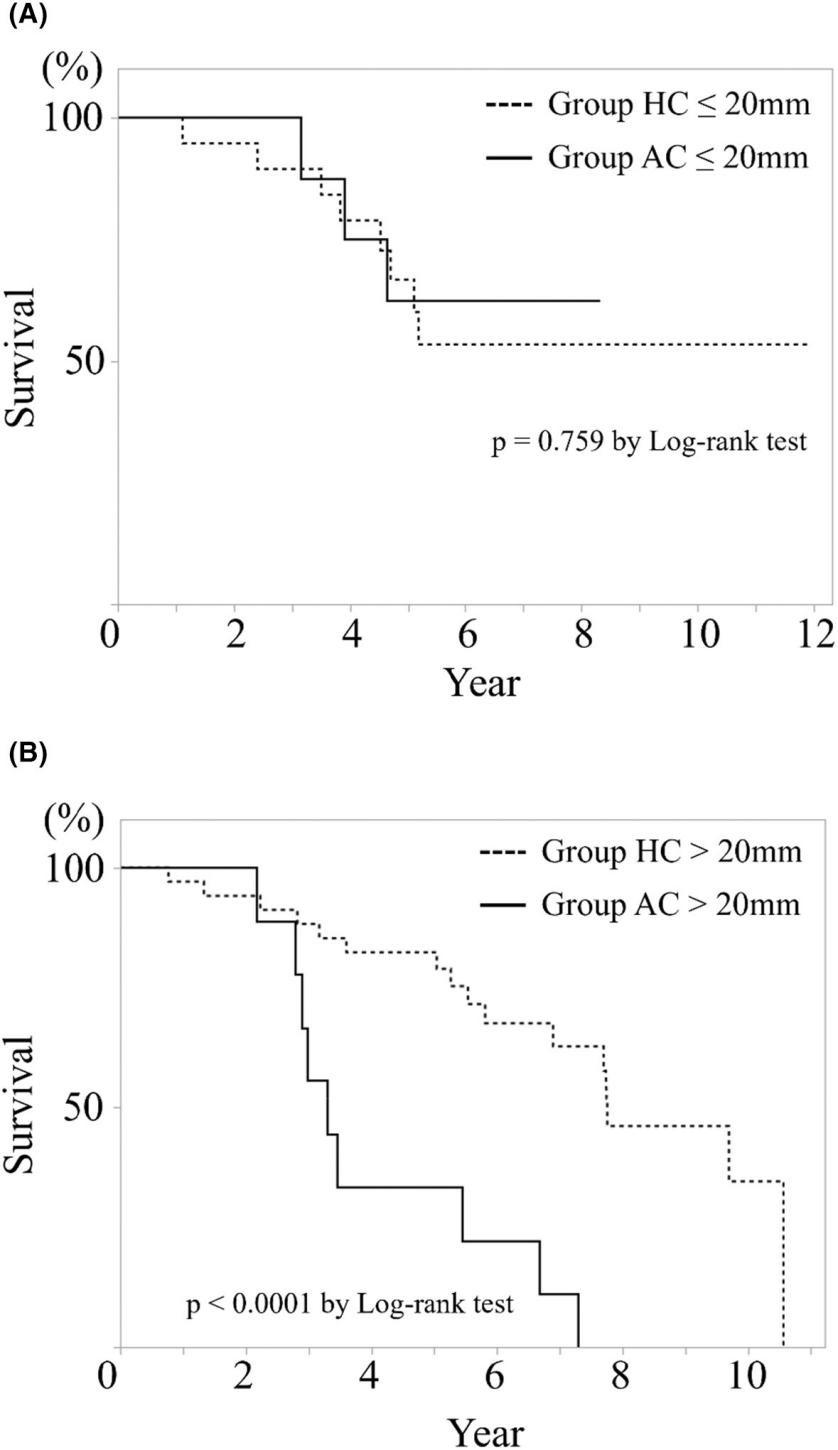
Overall survival after primary colorectal resection depending on colorectal liver metastasis size. Kaplan–Meier curve of (A) is ≤20 mm, and that of (B) is >20 mm There was a significant difference in prognosis after primary colorectal resection at >20 mm between two group. However, there was no difference in prognosis at ≤20 mm.

**FIGURE 4:**
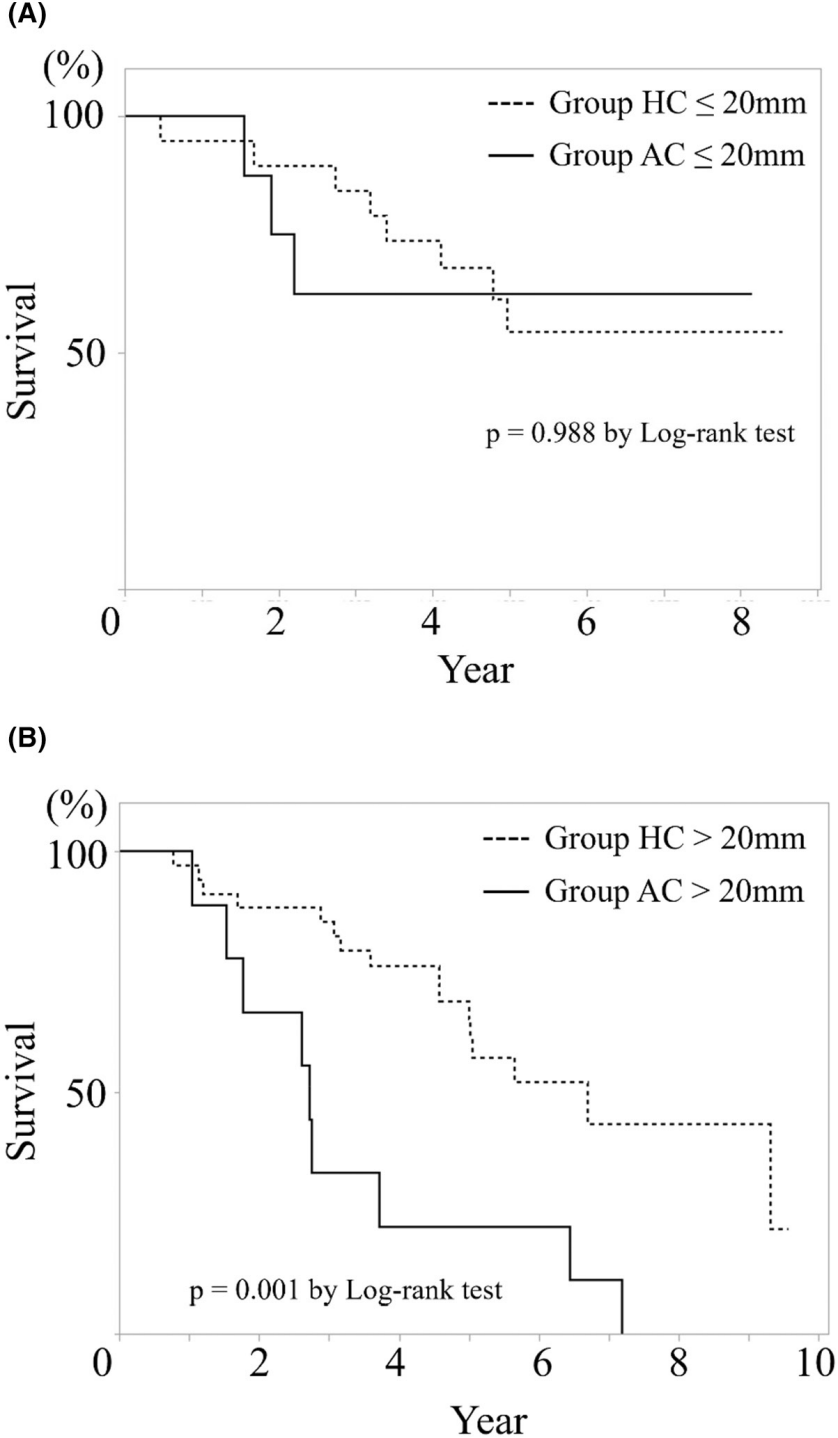
Overall survival after interventions for colorectal liver metastasis depending on liver tumor size. Kaplan–Meier curve of (A) is ≤20 mm, and that of (B) is >20 mm. There was a significant difference in prognosis after liver interventions at >20 mm between two group. However, there was no difference in prognosis at ≤20 mm.

**FIGURE 5:**
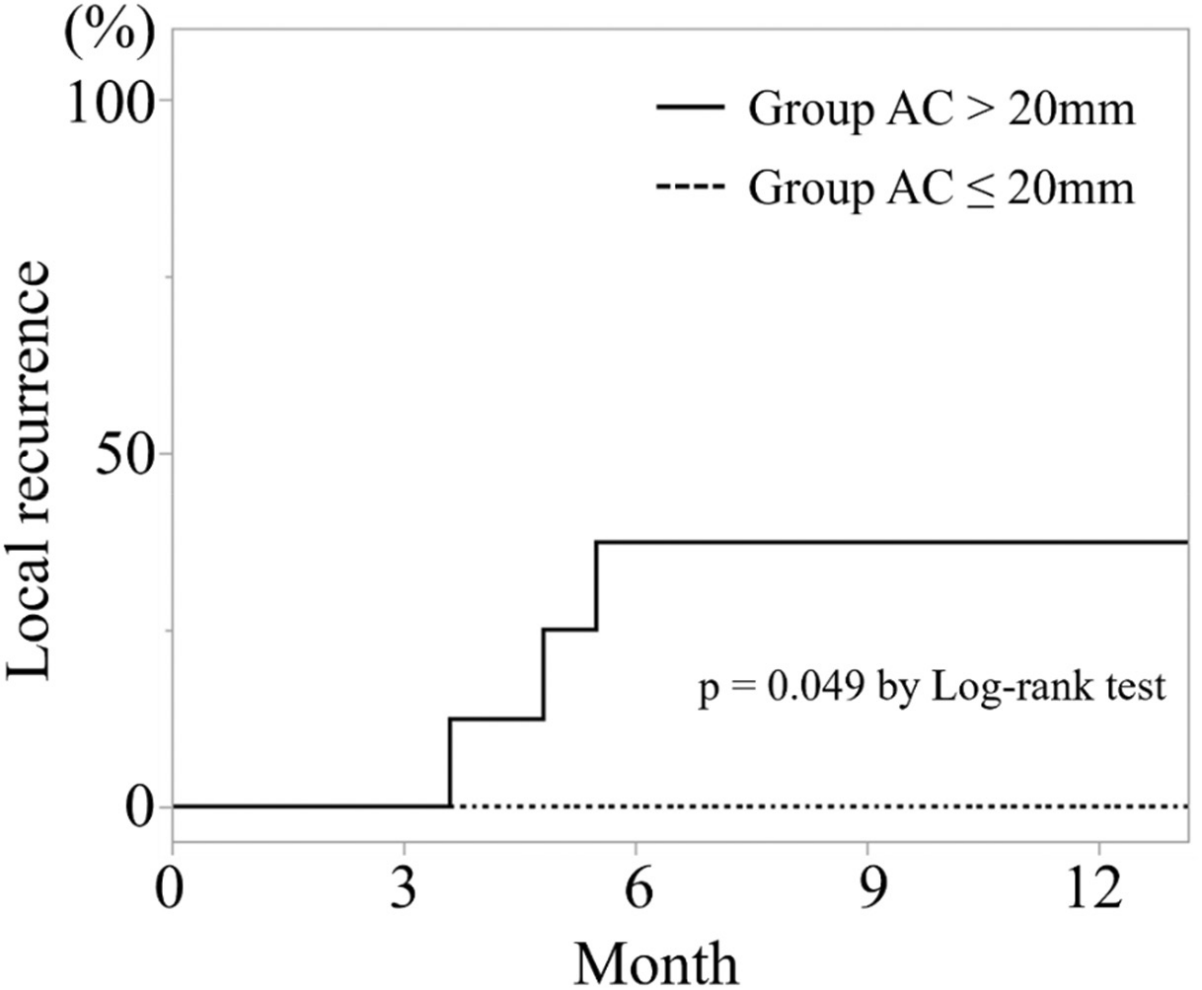
Local recurrence rate after ablations for colorectal liver metastasis depending on liver tumor size. There was a significant difference in local recurrence rate after liver ablations between >20 mm and ≤20 mm.

The overall survival, both after the primary colorectal resection and intervention for CRLM, was significantly worse in group AC, especially above the maximum tumor diameter of 20 mm (*p* < 0.0001, *p* = 0.001); there was no difference below 20 mm (Figures 3 and 4). Local recurrence after ablation for CRLM in ≤20 mm was not existent. However, there were three cases of recurrence in >20 mm. A significant difference was found between the two groups (*p* = 0.049) (Figure 5).

## 4 DISCUSSION

Percutaneous thermal ablation is less invasive than surgery. However, we cannot dismiss the possibility of residual viable lesions and local recurrences, as the lesion was not resected from the patient's body. The pathological diagnosis could not be confirmed by ablation without a preoperative tumor biopsy. Therefore, hepatectomy is often prioritized over ablation for the treatment of malignant tumors. As liver metastases are systemic, systemic chemotherapy is often prioritized over localized interventions for liver lesions. This makes it difficult to demonstrate the effectiveness of ablation for liver metastasis. We sometimes experience complete cure and long‐term survival in patients with advanced cancer with liver metastasis, having several types of treatments, including ablation. However, predicting a good clinical course before treatment is difficult.

Ablation for liver metastases at our department is performed for a variety of cancer types rather than hepatectomy. Localized interventions, including hepatectomy and ablation, have been reported for liver metastases from breast cancer.^9^ We performed ablation for 3 cm liver metastases in one patient with breast cancer. In this case, it was difficult to continue systemic chemotherapy because of its adverse effects. The patient was young and hoped to undergo aggressive treatment. After comprehensive discussion with the breast surgeon, the patient underwent ablation. The patient had no recurrence and had a tumor‐free period of 2 years. However, bone metastasis recurred. We did not know whether ablation contributed to her prognosis. We also encountered long‐term survivors of ablation for liver metastases of gastric cancer. However, we also encountered cases in which ablation did not contribute to an improved prognosis. We discussed individual cases and determined indications; however, we must perform ablation without absolute certainty. Therefore, we need to develop an evidence and criteria that reflect many patient factors.

The hepatectomy was more useful for CRLM than the ablation in our overall cases, but there was no difference in cases with tumor size ≤2 cm. Oshowo et al. reported that ablation and hepatectomy were equivalent for solitary CRLM.^10^ However, their report involved a small cohort, and such reports are rare. Several reports previously suggest that the hepatectomy is superior to the ablation for CRLM.^11^, ^12^, ^13^ Hepatectomy has been reported to be superior to ablation, even in solitary CRLM of <3 cm. The ablation for hepatocellular carcinoma is generally indicated in ≤3 cm tumor sizes. Since metastatic liver cancer has a higher biological malignancy potential than hepatocellular carcinoma, there should be a sufficient safety margin for ablation. We hypothesized that smaller tumor size would be a suitable indication for ablation. Our study demonstrated that hepatectomy and ablation were equivalent for CRLM <2 cm in size. Lee et al. also reported that ablation for ≤2 cm CRLM is suitable.^14^ This is the same view as that observed in our study. Ablation of a large CRLM may be avoided as much as possible. Ablation can replace hepatectomy for CRLM measuring <2 cm. A systematic review by Meijerink et al. reported that adding RFA improved the prognosis of patients compared to chemotherapy alone.^15^ Because adjuvant ablation for CRLM may be useful, its indications must be discussed. It should be noted that ablation is useful for CRLM. However, the criteria for applying ablation to CRLM are unclear. A prospective trial (COLLISION trial) is underway to demonstrate whether percutaneous thermal ablation is non‐inferior to hepatectomy for CRLM.^6^ This trial was registered for tumors <3 cm in size. We hope to perform a sub‐analysis of tumors <2 cm in size.

Surgical hepatectomy is the preferred choice for large tumors in our hospital. In contrast, ablation was performed for repeated recurrences at different time points. Ablation was performed in elderly patients compared with hepatectomy. This makes sense from the perspective of the invasiveness of each intervention. Lower serum albumin levels in group A indicated poorer nutrition than in group H. This finding suggests that patients with poor nutrition choose ablation over surgery. It was difficult to determine the lower platelet counts in group A because they were not so low to prevent surgery. This might reflect a decline in bone marrow function, or progression of liver fibrosis. It is known that platelet counts decrease with aging.^16^ In our study, there was no difference in chemotherapy between the two groups; however, the decline in bone marrow function may have been more pronounced in the elderly patients. This suggests that ablation was more easily adapted in our hospital, even when the patient was unsuitable for surgery.

The criteria for selecting appropriate cases for ablation must be primary cancer, patient's condition, chemotherapy, and size and number of liver lesions without a physician's unconscious bias. The strategy used at our hospital is hepatectomy as the first choice and ablation as the second choice. We believe that this strategy is similar for all other hospitals. Therefore, ablation tends to be performed on patients without surgical tolerance or suitability. There may have been physicians' unconscious biases. In our study, a tumor size of ≤2 cm should be a good indication for ablation of CRLM. It is necessary to discuss indications that include not only size but also age, general condition, and nutritional status. We hope that this study will help establish better criteria or indications for ablation of liver metastases.

There are several limitations in our study. First, this was a retrospective study. Further validation studies are required to confirm our results. Second, the sample size was small. However, there were various treatment factors, including surgery for the primary cancers, chemotherapy, and intervention for liver metastases, and the follow‐up period was long. Therefore, detailed investigation at multiple institutions was not easy. There were not many cases with liver metastases that could be treated with hepatectomy or ablation. These makes this field difficult to clarify.

## 5 CONCLUSIONS

Compared with hepatectomy, percutaneous thermal ablation has been performed for many cancer types. It is performed in elderly patients who have disadvantages in terms of blood tests. Ablation for CRLM sized ≤2 cm is a suitable indication with regard to overall survival. We suggest that ablation for colorectal liver metastases is necessary when carefully considering the maximum tumor diameter.

## AUTHOR CONTRIBUTIONS

**Seiichi Tawara:** Formal analysis (lead); investigation (lead); methodology (lead); project administration (lead); supervision (lead); writing – original draft (lead); writing – review and editing (equal). **Tetsuro Miyazaki:** Investigation (supporting). **Ryosuke Kiyota:** Investigation (supporting). **Yuki Maegawa:** Investigation (supporting). **Takeshi Shimizu:** Investigation (supporting). **Takuo Yamai:** Investigation (supporting). **Shoichiro Kawai:** Investigation (supporting). **Takuya Inoue:** Investigation (supporting). **Hisateru Komatsu:** Resources (supporting). **Akira Tomokuni:** Resources (supporting). **Masaaki Motoori:** Resources (supporting). **Takayuki Yakushijin:** Project administration (equal); supervision (equal); writing – review and editing (supporting).

## CONFLICT OF INTEREST STATEMENT

No conflict of interest exist.

## ETHICS STATEMENT

This was a retrospective study. This study was conducted in accordance with the Declaration of Helsinki, as amended in 2002. Patient information was anonymized; therefore, it could not be identified. We handled the data only in our department's information processing room at the Osaka General Medical Center. We have publicized the information about this study on our hospital's homepage. This clinical study was approved by the Institutional Review Board of the Osaka General Medical Center (No. 30‐S12‐002, accepted March 4, 2020).

## Data Availability

The data that support the findings of this study are available on request from the corresponding author. The data are not publicly available due to their containing information that could compromise the privacy of research participants.
